# Differential Cytokine Utilization and Tissue Tropism Results in Distinct Repopulation Kinetics of Naïve vs. Memory T Cells in Mice

**DOI:** 10.3389/fimmu.2019.00355

**Published:** 2019-03-04

**Authors:** Hye Kyung Kim, Hyunsoo Chung, Juntae Kwon, Ehydel Castro, Christopher Johns, Nga V. Hawk, SuJin Hwang, Jung-Hyun Park, Ronald E. Gress

**Affiliations:** ^1^Experimental Transplantation and Immunology Branch, Center for Cancer Research, National Cancer Institute, National Institutes of Health, Bethesda, MD, United States; ^2^Experimental Immunology Branch, Center for Cancer Research, National Cancer Institute, National Institutes of Health, Bethesda, MD, United States

**Keywords:** cytokines, apoptosis, migration, lymphopenia, proliferation

## Abstract

Naïve and memory T cells co-exist in the peripheral T cell pool, but the cellular mechanisms that maintain the balance and homeostasis of these two populations remain mostly unclear. To address this question, here, we assessed homeostatic proliferation and repopulation kinetics of adoptively transferred naïve and memory T cells in lymphopenic host mice. We identified distinct kinetics of proliferation and tissue-distribution between naïve and memory donor T cells, which resulted in the occupancy of the peripheral T cell pool by mostly naïve-origin T cells in short term (<1 week), but, in a dramatic reversal, by mostly memory-origin T cells in long term (>4 weeks). To explain this finding, we assessed utilization of the homeostatic cytokines IL-7 and IL-15 by naïve and memory T cells. We found different efficiencies of IL-7 signaling between naïve and memory T cells, where memory T cells expressed larger amounts of IL-7Rα but were significantly less potent in activation of STAT5 that is downstream of IL-7 signaling. Nonetheless, memory T cells were superior in long-term repopulation of the peripheral T cell pool, presumably, because they preferentially migrated into non-lymphoid tissues upon adoptive transfer and additionally utilized tissue IL-15 for rapid expansion. Consequently, co-utilization of IL-7 and IL-15 provides memory T cells a long-term survival advantage. We consider this mechanism important, as it permits the memory T cell population to be maintained in face of constant influx of naïve T cells to the peripheral T cell pool and under competing conditions for survival cytokines.

## Introduction

The peripheral T cell pool comprises a mixed population of recent thymic emigrants (RTE), naïve T cells and memory T cells, which all depend on IL-7 for survival and homeostasis (1–4). While the RTE and naïve T cell pool is constantly replenished by newly generated T cells from the thymus (5), most memory T cells are thought to have limited renewal capacities (6). Consequently, memory T cells face steep competition with newly arriving RTEs and pre-existing naïve T cells for IL-7-dependent signals to survive (7). IL-7 is a critical survival factor that upregulates anti-apoptotic Bcl-2 and Mcl-1 (8, 9), and it also promotes expression of trophic factors that are essential for T cell survival (10). The non-redundant requirement for IL-7 is illustrated in the severely compromised thymopoiesis of IL-7-deficient mice, and impaired survival of mature T cells in the absence IL-7 signaling (11, 12). Importantly, IL-7 is not produced by T cells so that T cells depend on exogenous IL-7 to survive. IL-7 is primarily produced by stromal cells and dendritic cells, and its expression is thought to be constitutive and developmentally set (13). Consequently, IL-7 availability constrains the size of the peripheral T cell pool (2, 14, 15). How the diversity and integrity of individual T cell subpopulations can be maintained in face of such competition is an intriguing question that has remained largely unresolved.

IL-7 signaling is transduced by the IL-7Rα and γc-chain complex (16). While expression of γc is considered to be constitutive (17), IL-7Rα expression is dynamically controlled during T cell development and differentiation (15, 18). Memory phenotype T cells express higher levels of IL-7Rα compared to naïve T cells (19), and it has been proposed that increased IL-7Rα expression would provide increased survival signals during effector to memory transition (20). Increased IL-7Rα expression is also utilized as a marker to identify memory precursor populations during an immune response (21). On the other hand, IL-7 signaling downregulates expression of its own receptor so that decreased IL-7Rα expression does not necessarily indicate less efficient IL-7 signaling (15). Thus, it remains unclear whether memory cells would utilize IL-7 more efficiently compared to naïve T cells. It is also not known how composition of the peripheral T cell pool is maintained when both memory and naïve T cells compete for the same resources to survive. Understanding these aspects of T cell homeostasis, however, has wide-ranging implications, and particularly in clinical settings of adoptive cell transfer (ACT) in cancer treatment or in T cell reconstitution after immune ablative procedures (22, 23). Studies in mice have shown that ACT into lymphopenic hosts strongly induces expansion of donor T cells *Ex vivo* expanded tumor infiltrating lymphocytes (TILs) into cancer patients was reported to better engraft in conjunction with a lympho-depleting regimen that creates lymphopenia (24). Moreover, depending on the differentiation status of donor T cells, such as naïve vs. memory or effector T cells, their anti-tumor activity, cytokine secretion and host grafting widely differed. The cellular and molecular basis of such distinct outcomes are still unresolved, but they remain of great interest to both clinicians and basic immunologists alike.

Here, we addressed these questions using mouse models of ACT, where distinct subsets of donor T cells were adoptively transferred into lymphopenic host mice and then monitored for their proliferation and expansion. Specifically, we examined competition of co-transferred naïve and memory T cells during IL-7-driven lymphophenia-induced homeostatic proliferation (25–27). Interestingly, short-term adoptive transfer (1 week) resulted in a preferential expansion and accumulation of naïve-origin T cells in the LN, so that they vastly outnumbered memory-origin T cells. Surprisingly, we found that such selective expansion of naïve T cells was limited to lymph nodes where IL-7 is abundant (13). In other organs, and specifically in non-lymphoid tissues, however, memory-origin donor T cells outnumbered naïve-origin donor T cells, indicating tissue-specific expansion of naïve vs. memory donor T cells. Mechanistically, we found that memory T cells were significantly less efficient to utilize and transduce signaling by IL-7, but that their ability to co-utilize IL-7 and IL-15 as homeostatic cytokines endows memory cells a competitive edge in their expansion over naive-origin T cells. Thus, memory T cells outcompete naïve T cells upon ACT into lymphopenic environments, and this process is controlled by their distinct utilization of homeostatic cytokines.

## Results

### Lymphopenia-Induced Homeostatic Proliferation of Naïve and Memory T Cells

**Figure 1:**
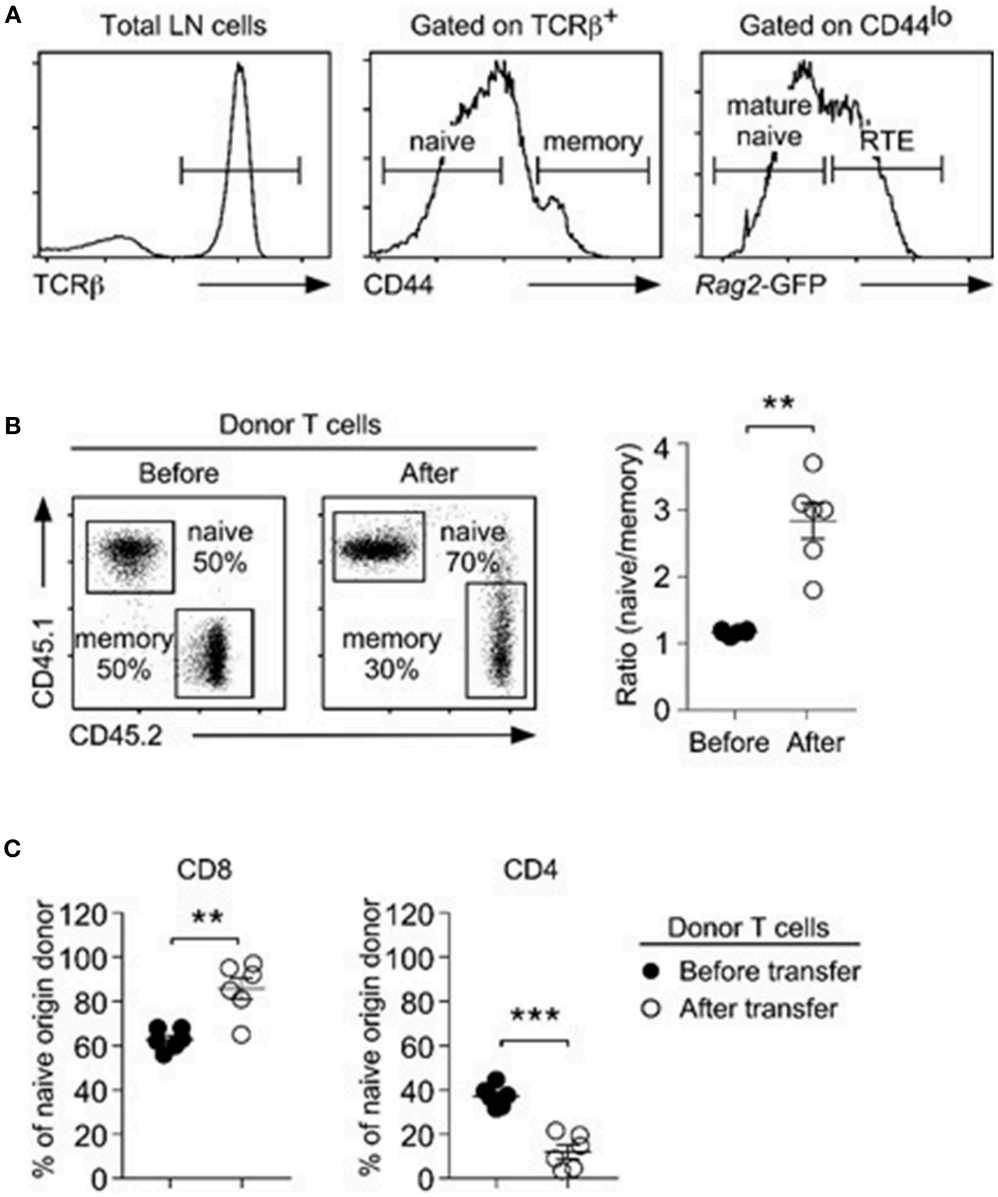
Naïve T cells outcompete memory T cells during lymphopenia-induced homeostatic proliferation. **(A)** Three distinct T cell subpopulations were identified in LN of *Rag2*-GFP reporter mice, based on surface CD44 and intracellular *Rag2*-GFP expression. CD44^hi^ cells corresponded to memory T cells (middle). Among CD44^lo^ T cells, *Rag2*-GFP^hi^ cells were considered recent thymic emigrants (RTE), and *Rag2*-GFP^neg^ cells were considered as naïve T cells. **(B)** Naïve (CD45.1) and congenic memory (CD45.2) T cells were injected into *Rag2*-deficient hosts and recovered 5 days later from the LN. Dot plots show representative distribution (left) and graph shows naïve vs. memory cell ratio (right) before and after injection. Data are summary of 6 independent experiments. **(C)** CD4 vs. CD8 ratio among naïve-origin donor T cells. Results show summary of 6 independent experiments. ^**^P < 0.01; ^***^P < 0.001.

In this study, we defined T cells expressing large amounts of CD44 (CD44^hi^) as memory T cells (28), while T cells with low abundance of CD44 (CD44^lo^) are considered as naïve T cells. We previously demonstrated that naïve T cells contain a significant fraction of RTE, which are functionally distinct to truly mature naïve T cells (7). Consequently, a mixed population of RTE and naïve T cells cannot correctly represent the survival kinetic of naïve T cells. Thus, we used the *Rag2*-GFP transgene (Tg) to identify truly mature naïve T cells (29), and only considered *Rag2*-GFP^neg^ CD44^lo^ T cells as naïve phenotype cells (Figure 1A). To examine homeostatic expansion of naïve and memory T cells under competing conditions, next, we purified naïve and memory T cells, mixed them at 1:1 ratio, and then injected them into lymphopenic (*Rag2*^−/−^) host mice. Naïve- vs. memory-origin donor T cells were identified using CD45.1/2 congenic markers. After 5 days, we recovered donor cells from host lymph nodes (LNs) for further analysis. Here, we were observed preferential accumulation of naïve-origin T cells, which resulted in dramatically increased naïve/memory ratios (Figure 1B). Among the donor T cells, we further found a selective increase in CD8 T cell frequencies that was concomitant to a decrease in CD4 T cell frequencies (Figure 1C), because CD4 donor T cells failed to undergo effective proliferation (Supplemental Figure 1A). These findings agree with previous observations that CD8 T cells expand more vigorously than CD4 T cells under lymphopenic conditions (30–32). Collectively, these results indicate that naïve T cells are superior to memory T cells in repopulating the T cell pool.

### Accelerated Proliferation of Memory T Cells Under Lymphopenic Conditions

**Figure 2:**
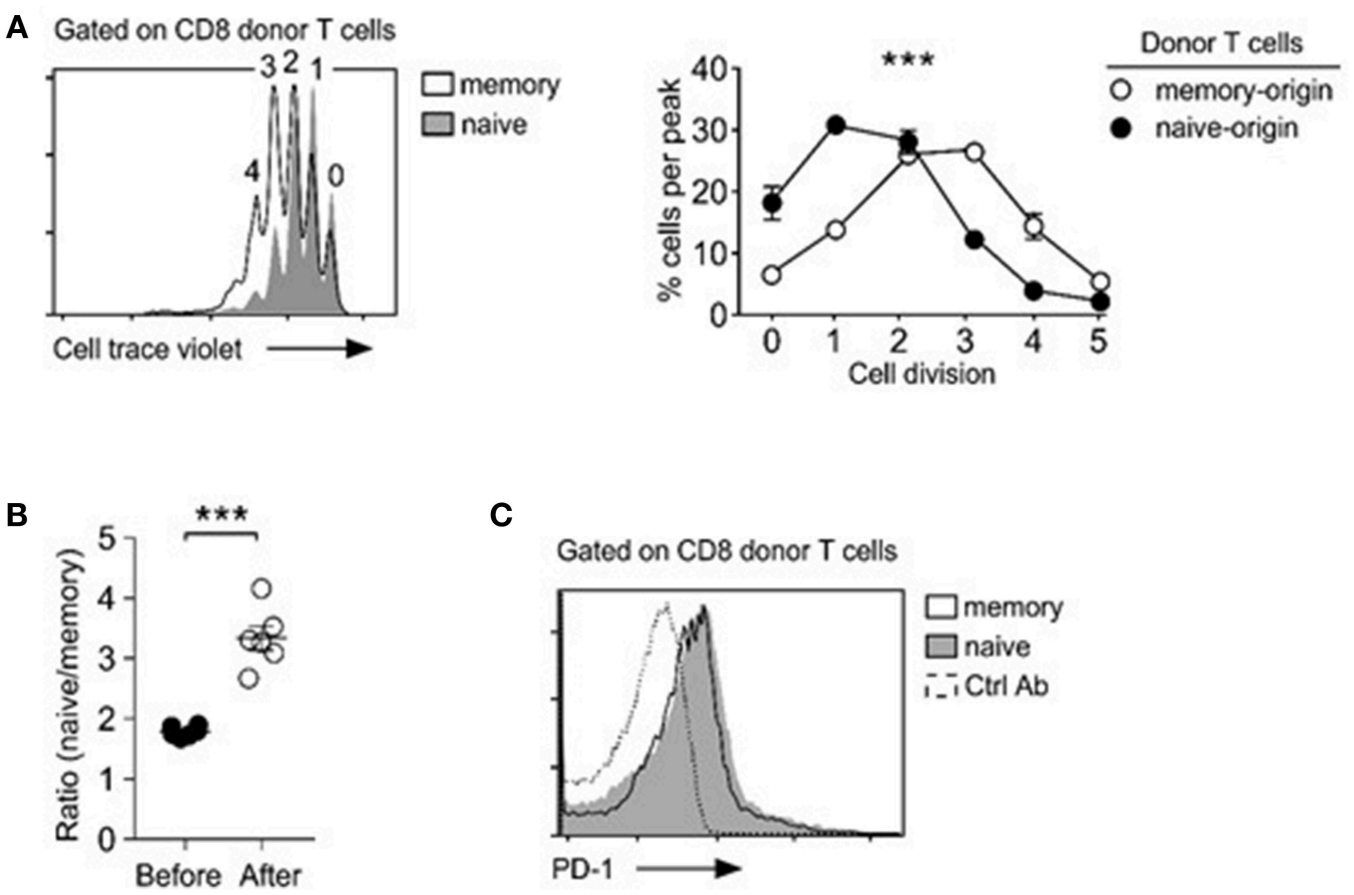
Memory T cells outpace naïve T cells in lymphopenia-induced homeostatic proliferation. **(A)** Cell trace violet dilution of naïve- and memory-origin donor CD8 T cells. Cell proliferation in *Rag2*-deficient host mice (*Rag2*^−/−^) was determined by assessing percentage of cells in individual cell trace violet peaks (left). Cells that underwent no or one division (0, 1) were considered as resting cells. Cells that have undergone more than two divisions (>2) were considered as proliferating cells (bottom). Data are shown as mean ± SEM (*n* = 8 mice) and represent summary of 4 independent experiments. *P*-values were determined by two-way ANOVA. **(B)** Naïve vs. memory-origin CD8 T cell ratio before and after adoptive transfer into lymphopenic *Rag2*-KO mice. Data are summary of six experiments. **(C)** Surface PD-1 expression on naïve and memory T cell origin donor T cells in LN (left) and spleen (right) of host mice after 5 days of adoptive transfer. Histograms are representative of 2 independent experiments. ^***^P < 0.001.

To gain mechanistic insights into the distinct repopulation efficiencies, we examined proliferation of naïve- vs. memory-origin CD8 T cells. To this end, we purified naïve and memory T cells and labeled them with Cell Trace Violet (CTV) before their adoptive transfer. Dilution of an intracellular dye such as CTV can serve as a faithful marker of proliferation, and thus accurately reports the proliferative history of a given cell population (33). Surprisingly, and contrary to our expectation, we found that memory CD8 T cells proliferated substantially faster than naïve T cells (Figure 2A), which resulted in increased naïve/memory CD8 T cell ratio after adoptive transfer (Figure 2B). Thus, while memory T cells undergo more vigorous proliferation than naive T cells, paradoxically, memory donor T cells did not outnumber donor naïve T cells after homeostatic proliferation.

T cell exhaustion is a homeostatic mechanism that trims the size of the activated memory T cell pool (34). PD-1 is a marker for T cell exhaustion (35), and we wished to determine if the rapid and excessive proliferation could induce T cell exhaustion in adoptively transferred memory T cells. Surface analysis for PD-1 expression, however, did not show noticeable difference between memory and naïve CD8 donor T cells (Figure 2C). We also did not see increased caspase-3 activity in memory T cells (data not shown). Collectively, these results indicate that exhaustion or increased cell death are unlikely causes for inefficient expansion of memory T cells in adoptive transfer experiments.

### Diminished IL-7 Signaling in Memory T Cells

**Figure 3:**
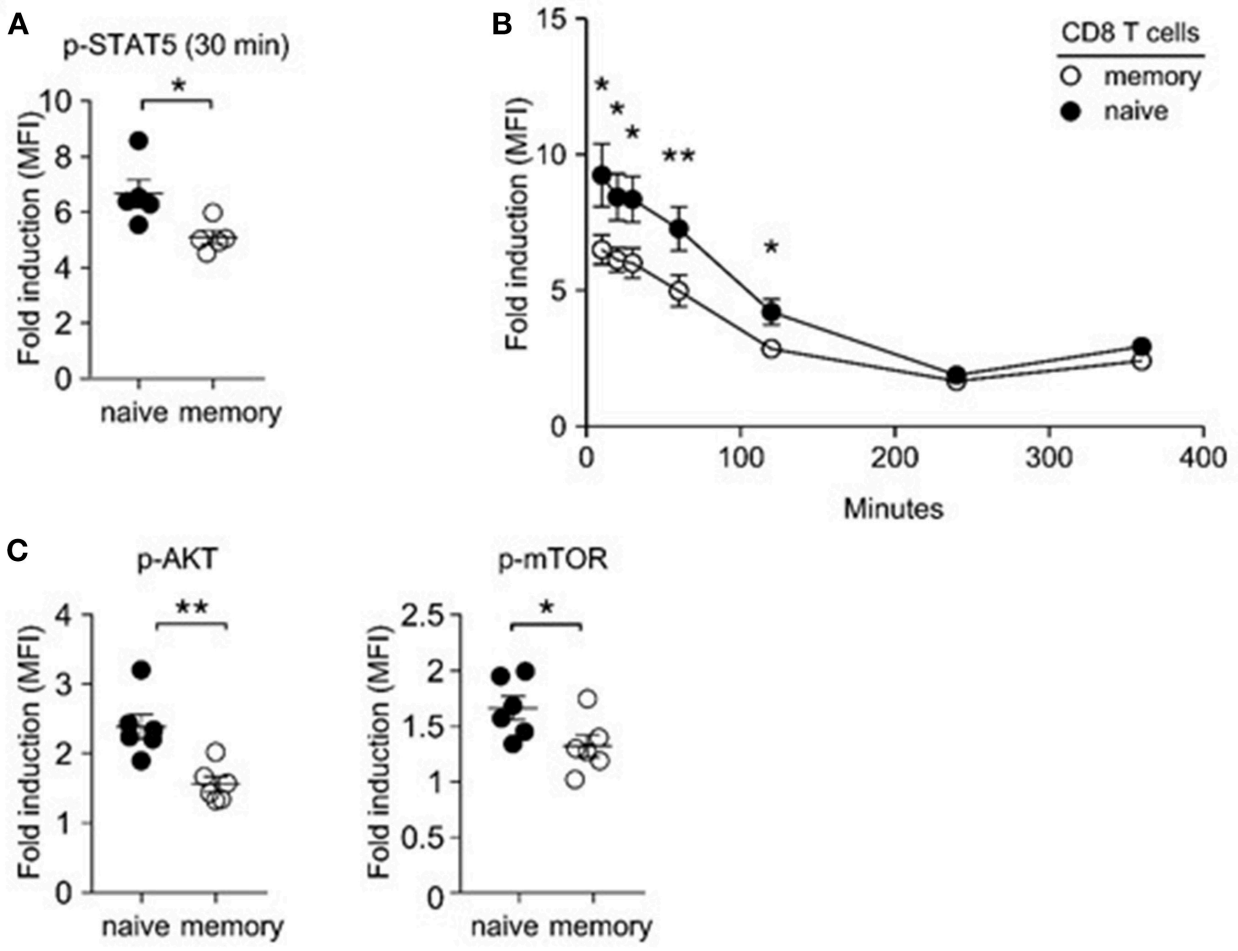
Impaired IL-7 signaling in memory CD8 T cells. **(A)** IL-7-induced STAT5 phosphorylation in naïve and memory CD8 T cells. Freshly isolated LN cells were stimulated with IL-7 (1 ng/ml) for 30 min and assessed for phosphor-STAT5 content in naïve and memory CD8 T cells. Graph shows summary of 5 independent experiments. **(B)** Kinetics of IL-7 (1 ng/ml)-induced STAT5 phosphorylation in naïve and memory T cells at 10, 20, 30, 60, 120, 240, and 360 min of stimulation. Graph shows summary of two independent experiments. **(C)** IL-7-induced phosphorylation of Akt and m-TOR in naïve and memory T cells. Graphs show summary of 6 independent experiments. ^*^P < 0.05; ^**^P < 0.01.

Lymphopenia-induced homeostatic proliferation depends on IL-7 signaling (36). Thus, we wished to know if memory T cells would be less efficient in IL-7 signaling, which could result in their impaired expansion and accumulation upon adoptive transfer. To this end, we examined IL-7-induced STAT5 phosphorylation in naïve and memory CD8 T cells. Compared to naïve T cells, memory T cells were substantially blunted in their IL-7 response, as demonstrated in significantly reduced amounts of phosphorylated STAT5 (pSTAT5) relative to that of naïve CD8 T cells (Figure 3A). Detection of intracellular pSTAT5 was highly specific, because IL-7 signaling in STAT5-deficient T cells did not show any pSTAT5 activity in the same assay (Supplemental Figure 1B). To exclude a difference in signaling kinetics, we monitored pSTAT5 contents at early time points (10, 20, and 30 min) and also after prolonged IL-7 stimulation (2 and 4 h), and still found both CD4 and CD8 memory T cells being significantly blunted in their IL-7 response compared to naïve T cells (Figure 3B, Supplemental Figure 1C). Such reduced IL-7 responsiveness further translated into diminished downstream effector molecule activation, so that Akt and mTOR phosphorylation were significantly decreased in IL-7-signaled memory CD8 T cells compared to naïve CD8 T cells (Figure 3C). Akt and mTOR are serine kinases that upregulate T cell metabolism and provide anti-apoptotic signals (37). Consequently, we considered the possibility that suboptimal IL-7 signaling in memory T cells could result in increased cell death, which would lead to a preferential loss of memory T cells in mixed donor T cell adoptive transfer experiments.

### Increased Pro-survival Factor Expression in Memory T Cells

**Figure 4:**
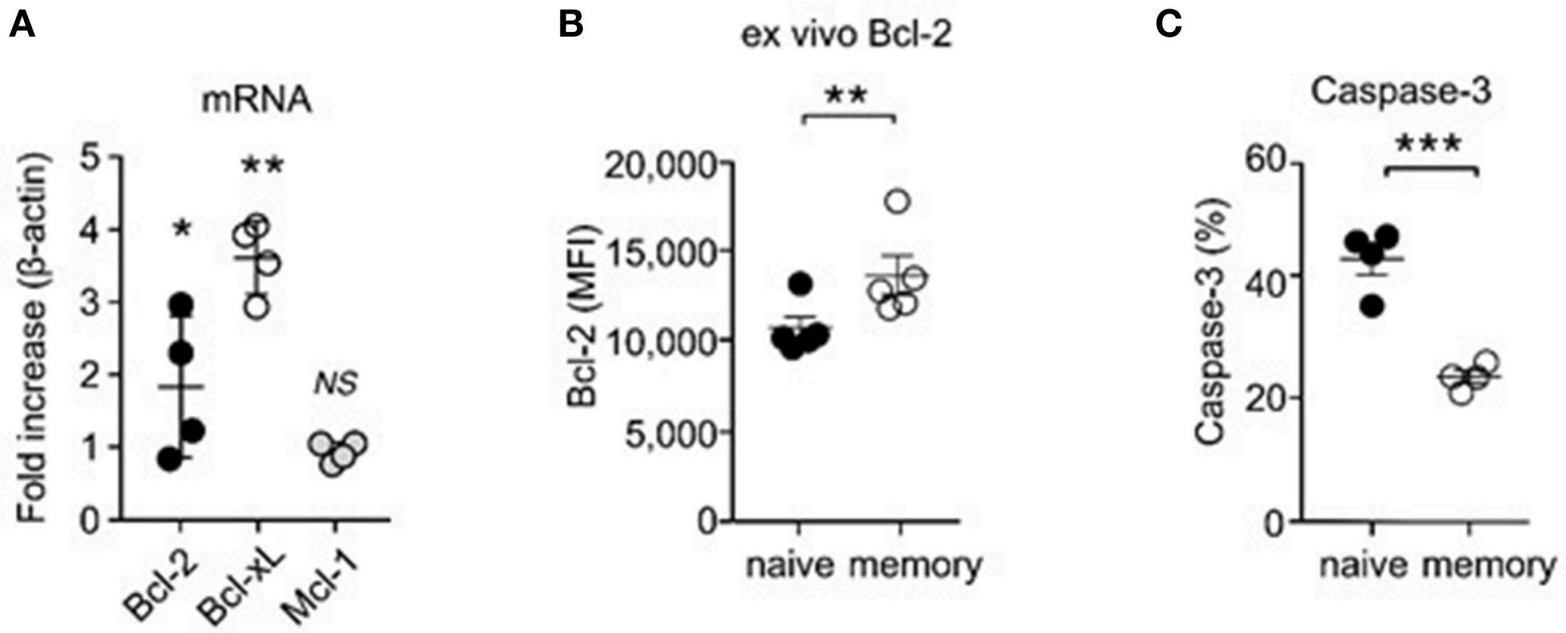
Increased pro-survival molecule expression and enhanced survival of CD8 memory T cells. **(A)** Electronically sorted naïve and memory CD8 T cells were assessed for mRNA expression of Bcl-2, Bcl-x_L_, and Mcl-1. Data are summary of 4 independent experiments. **(B)** Intracellular staining for Bcl-2 proteins in naïve and memory CD8 T cells. Graph shows summary of 5 independent experiments. **(C)** Active caspase-3 contents in naïve and memory CD8 T cells. Data are summary of 5 independent experiments. ^*^P < 0.05; ^**^P < 0.01; ^***^P < 0.001.

To examine if memory T cells would be more prone to apoptosis, we assessed expression of survival molecules in naïve and memory CD8 T cells. Bcl-2 is a major anti-apoptotic molecule downstream of IL-7 (8). We expected that memory T cells would express significantly smaller amounts of Bcl-2 than naïve T cells, because we found memory CD8 T cells to show decreased IL-7 responsiveness. Strikingly, and contrary to our expectation, qRT-PCR analysis revealed that memory T cells expressed significantly larger amounts of Bcl-2 mRNA transcripts than naïve T cells (Figure 4A), which further correlated with increased Bcl-2 protein expression (Figure 4B). In agreement, assessing the intracellular contents of active caspase-3, which is a measure of apoptosis (38), revealed that memory T cells were less apoptotic than naïve T cells (Figure 4C). Increased cell survival, however, would contradict our finding that memory T cells are less effective in IL-7 signaling than naïve T cells. To solve this conundrum, we examined expression of other pro-survival molecules, and we noted that Bcl-x_L_ mRNA expression was highly upregulated in memory T cells compared to naïve T cells (Figure 4A). Bcl-x_L_ is a potent anti-apoptotic protein that is induced by IL-15 signaling (39–42). IL-15 utilizes the IL-2Rβ/γc cytokine receptor complex for signaling (16, 43), and memory T cells, but not naïve T cells, express large amounts of IL-2Rβ (44). Therefore, these results suggest that the ability to co-utilize IL-15 together with IL-7 could provide survival and proliferative advantage to memory T cells over naïve T cells.

### IL-15 Signaling Promotes Proliferation of Memory T Cells

**Figure 5:**
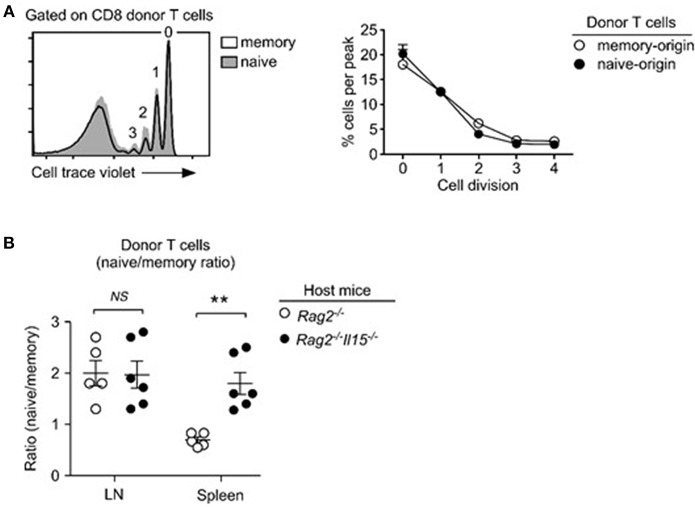
IL-15 drives the accelerated homeostatic proliferation of memory T cells. **(A)** Lymphopenia-induced homeostatic proliferation of naïve and memory CD8 donor T cells in *Rag2, IL15*-double-deficient (*Rag2*^−/−^*Il15*^−/−^) mice. Histogram shows representative CTV dilution of donor T cells (left). Cell proliferation in *Rag2*^−/−^*Il15*^−/−^ host mice was determined by assessing percentage of cells in individual cell trace violet peaks (right). Data are representative of 6 independent experiments. **(B)** Naïve- vs. memory-origin donor T cell ratio in LN and spleen of *Rag2*^−/−^ and *Rag2*^−/−^*Il15*^−/−^ host mice. Data are summary of 6 experiments. ^**^P < 0.01.

Based on these observations, we wished to know if IL-15 indeed provides additional proliferative cues to memory T cells. If this would be the case, it would explain how memory CD8 T cells can outpace naïve T cells in proliferation, even as they are less efficient in IL-7 signaling. To this end, we adoptively transferred a 1:1 mixed population of naïve and memory donor T cells into *Rag2*^−/−^*Il15*^−/−^ mice and assessed their proliferation. Interestingly, unlike in IL-15-sufficient *Rag2*^−/−^ hosts, memory T cells in IL-15-deficient *Rag2*^−/−^ (*Il15*^−/−^*Rag2*^−/−^) host mice did not proliferate more vigorously than naïve T cells, and we did not observe differences between naïve and memory T cell proliferation (Figure 5A). These results indicate that IL-15 is the driver of accelerated proliferation of memory-origin T cells during lymphopenia-induced homeostatic proliferation.

Consequently, when assessing the accumulation of naïve- vs. memory-origin donor T cells in lymph nodes (LN) of *Rag2*^−/−^*Il15*^−/−^ host mice, we did not notice any significant changes compared to IL-15-sufficient *Rag2*^−/−^*Il15*^+/+^ hosts (Figure 5B, left). Naïve-origin T cells still outnumbered memory-origin donor T cell after 1 week of adoptive transfer, regardless of the presence or absence of host IL-15. On the other hand, we found a dramatic change in the naïve- vs. memory-origin T cell ratio in the spleen, where naïve-origin T cells were significantly more abundant than memory-origin T cells (Figure 5B, right). Thus, the lack of host IL-15 significantly impaired the expansion of memory T cells in the spleen, but not in the LN. These results suggest that IL-15 would contribute to the expansion of memory T cells in LNs. In contrast, IL-15 is abundantly expressed in the spleen (Figure 5B) (45), and can act as a major contributor to memory T cell proliferation. In agreement, the naïve- vs. memory-origin ratio was reversed in the spleen, so that memory-origin T cells outnumbered naïve-origin T cells. These results reveal a previously unappreciated aspect of lymphopenia-induced homeostatic proliferation that is associated with tissue-specificity and differential usages of homeostatic cytokines.

### Distinct Tissue Migration of Adoptively Transferred Naïve and Memory T Cells

**Figure 6:**
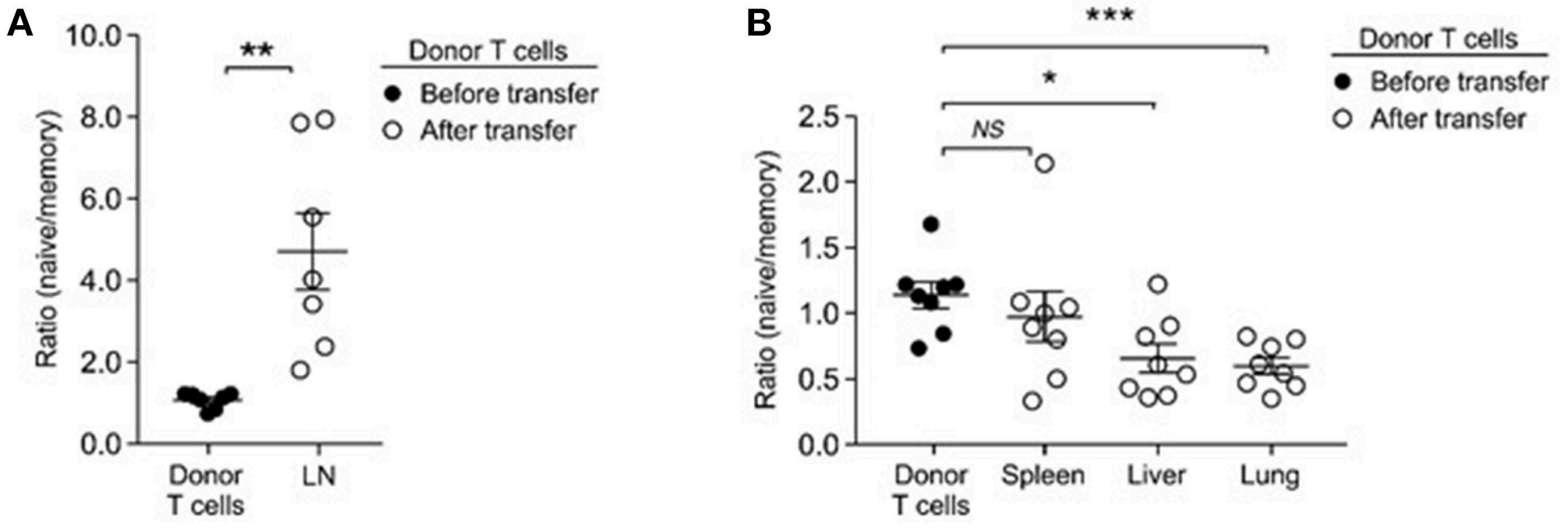
Memory T cells preferentially home to non-lymphoid tissues. **(A)** Naïve- to memory-origin T cell ratio among donor cells recovered from LN of *Rag2*^−/−^ host mice. Data are summary of seven experiments. **(B)** Naïve- to memory-origin T cell ratio among donor cells recovered from spleen, liver, lung of *Rag2*^−/−^ host mice. Data are summary of 7 experiments. ^*^P < 0.05; ^**^P < 0.01; ^***^P < 0.001.

While IL-15's contribution would explain the preferential accumulation of memory-origin T cells in the spleen, it remained unclear to us why naïve-origin T cells would outnumber memory-origin T cells in the LN upon homeostatic proliferation. As a potential explanation, we considered that adoptively transferred memory-origin donor T cells would be inefficient in seeding the LN. In fact, memory T cells display an activated phenotype that comprises downregulation of lymphoid tissue homing and retention molecules, such CD62L and CD103 (46, 47). Thus, relative to naïve T cells, memory T cells would be impaired or delayed in entering lymph nodes after adoptive transfer. Accordingly, we hypothesized that, depending on whether the donor T cells would be of naïve or memory T cell origin, T cells would migrate and occupy survival niches in different organs. To test this idea, we performed short-term transfer experiments where we injected a 1:1 mixture of naïve and donor T cells into lymphopenic host mice. We harvested donor T cells after 3 days, instead of the usual 5 days, of injection to monitor migration in the absence of proliferation. In addition to LN (Figure 6A), we harvested T cells from other organs, such as spleen, lung and liver, and examined the naïve/memory-origin donor T cell ratio in these tissues (Figure 6B). As expected, we found significant and preferential accumulation of naïve-origin T cells in the LN (Figure 6A). Other organs, however, were preferentially seeded with memory-origin T cells, indicating distinct tissue migration between naïve and memory T cells (Figure 6B). To demonstrate that the selective accumulation of naïve-origin T cells in LN was mediated by LN-specific adhesion molecules, next, we asked if memory T cells would also accumulate in LN if they would express tissue homing molecules, such as CD62L. Notably, among CD44^hi^ memory T cells, the central memory T cell population expresses large amounts of CD62L and differs from effector memory T cells that are absent for CD62L (Supplemental Figure 2A). Thus, we expected that central memory donor T cells would accumulate in LN as is the case for naïve donor T cells. This was precisely the case, as we found that effector memory donor T cells that lack CD62L were substantially outnumbered by naïve donor T cells in the LN, but that central memory donor T cells which express CD62L were found in similar ratios to naïve donor T cells in the LN (Supplemental Figure 2B). Importantly, both effector and central memory donor T cells proliferated more vigorously than naïve donor T cells (Supplemental Figure 2C), effectively excluding delayed proliferation as a basis of impaired accumulation of effector memory T cells. Collectively, these results suggested that memory T cells survive and accumulate as efficient as naïve T cells, but that their initial migration and accumulation differ among tissues in the host.

### Co-utilization of IL-15 and IL-7 Promotes Long Term Survival Advantage to Memory T Cells

**Figure 7:**
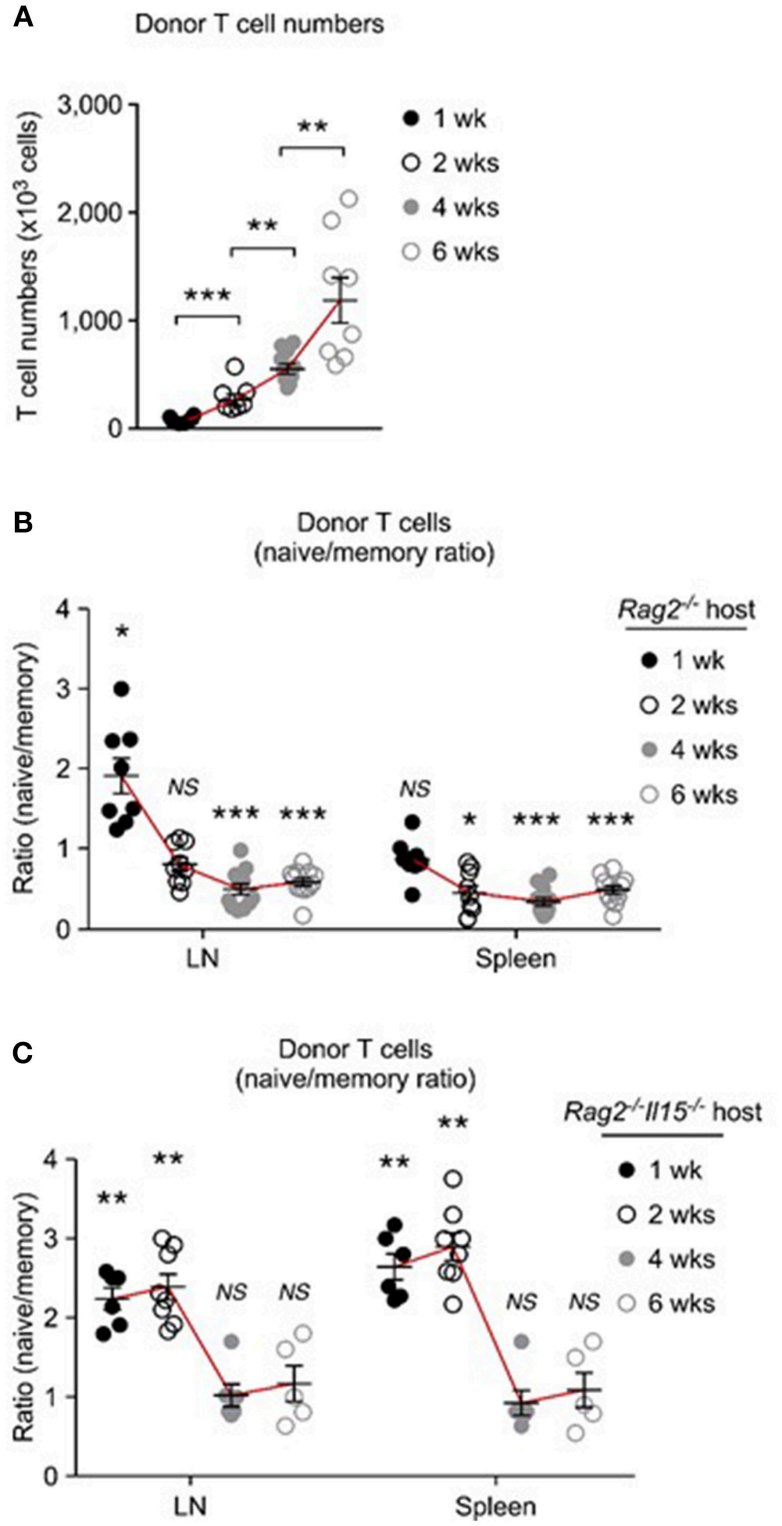
Distinct repopulation kinetics of naïve and memory T cells upon homeostatic proliferation. **(A)** Pooled donor T cell numbers from LN and spleen of host mice after adoptive transfer into *Rag2*-deficient mice. Graph shows result from each 6, 7, 10, and 8 experiments for 1 week, 2 weeks, 4 weeks, and 6 weeks, respectively. **(B)** Naïve to memory ratio among donor T cells recovered from spleen and LN of *Rag2*^−/−^ host mice. Graph shows result from each 8, 9, 12, and 12 experiments for 1 week, 2 weeks, 4 weeks, and 6 weeks, respectively. **(C)** Naïve to memory ratio among donor T cells recovered from spleen and LN of *Rag2*^−/−^*Il15*^−/−^ host mice. Graph shows result from each 6, 8, 6 and 5 experiments for 1 week, 2 weeks, 4 weeks, and 6 weeks, respectively. ^*^P < 0.05; ^**^P < 0.01; ^***^P < 0.001.

Because of such differences in homeostatic expansion among tissues, we considered that homeostatic expansion outside of the LN would result in accumulation of memory-origin T cells. This was indeed the case. To obtain a more comprehensive picture of donor T cell proliferation, first, we monitored T cell expansion beyond the short-term (5 days) adoptive transfer, and assessed donor T cells numbers at 1, 2, 4, and 6 weeks after injection. Strikingly, with increasing time of adoptive transfer, there was a dramatic increase in total donor T cell numbers that we could recover from LN and spleen of host mice (Figure 7A). However, it is important to point out that naïve- and memory-origin donor T cells accumulated in the host at unequal ratios. With increasing number of donor T cells, we found a preferential expansion of memory-origin T cells in both LN and spleen (Figure 7B). Initially, there was a preferential accumulation of naïve-origin donor T cells in the LN (Figure 7B, 1st week time point). However, such skewed expansion was limited to the first week of adoptive transfer, and it was not found after 2 weeks or thereafter, and not in any other organ (Figure 7B). Collectively, these results document a lagged response of memory-origin donor T cells in peripheral expansion, that is presumably driven by IL-15 in non-lymphoid tissues and outside of the LN.

To directly examine the role of IL-15 in this process, next, we performed the same adoptive transfer experiments into IL-15-deficient *Rag2*^−/−^*Il15*^−/−^ mice (Figure 7C). Strikingly, in the absence of host IL-15, the expansion and accumulation of memory-origin donor T cells were significantly delayed, and the preferential expansion of naïve-origin T cells continued for another week or more (Figure 7C). As shown in Figure 7C, adoptively transferred memory-origin T cells were still outnumbered by naïve-origin T cells up to 2 weeks and in both LN and spleen in *Il15*^−/−^*Rag2*^−/−^ host mice (Figure 7C). Eventually, memory-origin T cells caught up with naïve-origin T cells so that after 4 and 6 weeks of adoptive transfer, the naïve vs. memory-origin T cell ratio was reduced and we found them in similar frequencies (Figure 7C). Because we did not observe an effective expansion of memory-origin T cells after 6 weeks of transfer, these data further confirm the IL-15 requirement for effective memory T cell repopulation. Collectively, these results indicate that co-utilization of IL-7 and IL-15 significantly affects the initial expansion of memory T cells, and that it further provides long-term survival advantage to memory T cells during homeostatic proliferation.

## Discussion

Maintaining the memory T cell pool is a critical aspect in T cell immunology as it provides the reservoir for rapid and vigorous immune responses to re-challenging antigenic insults. Importantly, T cell memory is formed in the presence of antigens, but memory cells need to be maintained after clearance of pathogens. Therefore, memory T cells are thought to survive in the absence of TCR-mediated antigen stimulation and rather rely on homeostatic cytokines for their survival (48, 49). IL-7 is a key homeostatic cytokine for memory T cell survival, but its expression is scarce and limited to few tissues (13). Because RTEs and naïve T cells also require IL-7 signaling, competition for IL-7 has been proposed to be a mechanism to control the size of the peripheral T cell pool (7, 15). While the strict IL-7 dependency of each T cell subsets is well established, it is less well known how the competition among individual T cell populations would maintain the subset composition of the peripheral T cell pool.

As a potential solution, our current study reports that memory-origin donor T cells outpace naïve-origin donor T cells during homeostatic proliferation. Specifically, we identified IL-15 as the driver for fast proliferation of memory T cells under lymphopenic conditions. IL-7 drives the expansion of both naïve and memory T cells, but IL-15 has an additive effect on IL-7-mediated proliferation of memory T cells. Consequently, memory T cells have a proliferative advantage over naïve T cells once entering the peripheral T cell pool. The selective effect of IL-15 on memory T cells was imposed by the distinct expression of IL-2Rβ, which is necessary for binding and signaling of IL-15 on target cells (50). Memory cells express high levels of IL-2Rβ, but naïve T cells only express very low levels of IL-2Rβ and are thus inefficient to bind and signal IL-15 (51).

Despite the contribution of IL-15 to accelerate their proliferation, it was curious that memory donor T cells were less efficient than naïve donor T cells to repopulate the lymphopenic environment of *Rag2*-deficient mice. Initially, we considered the possibility that excessive proliferation would be detrimental for memory T cell survival such as by inducing exhaustion that would result in increased cell death. Analysis for surface PD-1 expression and intracellular caspase-3 activity, however, suggested that this was not the case. Therefore, we faced a conundrum that, despite increased proliferation, diminished numbers of memory-origin donor T cells were recovered from host mice compared to naïve origin T cells. Previously, we showed that short term administration of recombinant IL-7 proteins resulted in tissue-redistribution of naïve and RTE cells, so that RTE preferentially accumulated in lymphoid tissues (3). Analogous to distinct trafficking of RTE and naïve T cells, here, we considered the possibility that distinct tissue migration and tropism of naïve vs. memory-origin donor T cells could provide the molecular basis for impaired expansion of memory T cells, and our current data are in support of this idea.

Memory T cells are more agile and migratory than naïve T cells, which agrees with their prime mission to survey tissues for pathogenic antigens (21). Accordingly, naïve and memory T cells express different sets of chemokine receptors and cell adhesion molecules (52). Naïve T cells express large amounts of the chemokine receptor CCR7 and the cell adhesion molecule CD62L which facilitate their migration and entrance into secondary lymphoid tissues. Memory T cells, on the other hand, express CCR9 and CXCR3, which promote trafficking to peripheral tissues (53, 54). Moreover, memory T cells, but not naïve T cells, preferentially home to the bone marrow, where they undergo expansion (55), and homeostatic proliferation (56). As a corollary, adoptively transferred memory and naïve T cells could disperse to distinct tissues and establish residency and undergo homeostasis. In agreement with this idea, we found that adoptively transferred memory-origin donor T cells preferentially migrated into non-lymphoid tissues, such as liver and lung, but then gradually re-appeared in secondary lymphoid organs. After 2 weeks of adoptive transfer, memory-origin donor T cells then outnumbered naïve T cells also in the LN. Thus, the maintenance of the memory T cell pool is driven by a slow kinetic of expansion that is regulated by two homeostatic cytokines and will eventually outcompete and outnumber naïve T cells. This observation raises two important issues; First, short-term (<1 week) analysis of adoptive transfer, which is usually the method of choice in assessing lymphopenia-induced homeostatic proliferation, provides an inaccurate picture of the *in vivo* events of T cell repopulation. As such, discovering the delayed kinetic of memory T cell expansion was enlightening, because it revealed that inefficient recovery of memory donor T cells at early time-points was not due to their failure to expand. Instead, it was the inefficient recruitment of memory-origin donor T cells into LNs which made it appear as if naïve T cells would be superior in their repopulation of the lymphopenic environment. Secondly, despite their better responsiveness to IL-7, naïve T cells are intrinsically less efficient in repopulating a lymphopenic environment compared to memory T cells. Naïve-origin donor T cells are less proliferative, and their expansion is mostly limited to LN tissues. Consequently, the homeostasis of the naïve T cell pool depends on thymic output and the continuous influx of newly generated naïve T cells. Collectively, these findings showed that the T cell subset origin of donor T cells determines the engraftment efficacy, repopulation kinetics, and tissue-distribution of adoptively transferred T cells.

The clinical implications of this study are manifold, and they provide new insights into designing ACT in cancer immunotherapies as well as for understanding CD8 T cell-mediated GVHD upon allogeneic stem cell transplantations. Specifically, it has been a long-standing question which T cell subset would be the most effective in adoptive immunotherapies (57). There is a consensus emerging that attributes less-differentiated, naïve phenotype T cells being the subset with the greatest curative and anti-tumoral potential (22, 58, 59). The exact mechanism underlying this observation remains to be unraveled. However, multiple pathways have been proposed, such that undifferentiated T cells would possess greater proliferative potential, retain the ability to produce IL-2, and display greater anti-tumor efficacy (60, 61). In addition to these, it is also proposed that the lymphoid homing molecule L-selectin (CD62L), which is usually associated with a naïve phenotype promotes anti-tumor effects in ACT. Such propensity is illustrated in the superior effector function of naive T cells and central memory T cells both of which are marked by the expression of CD62L (62). On the other hand, there are also conflicting data about the role of CD62L in ACT (63), where the failure to express CD62L did not impair the function of donor T cells and did not alter the outcome of T cell adoptive immunotherapy in mice (63). Because CD62L expression is usually associated with a more undifferentiated phenotype, these results suggest that it is rather the cell intrinsic property than CD62L expression itself that confers superior function to the CD62L^+^ subset in ACT. Along these lines, it would be important to ensure that the repopulated T cell pool in ACT or after ablative immune would retain a naïve phenotype to maximize its function, and our data indicate this could be achieved by minimizing the incorporation of memory-origin donor T cells. Further, these data now provide a mechanistic understanding for our previous observation in humans that CD4^+^ naïve cells never reconstitute to baseline levels without thymic recovery (64)

These data also further reinforce the importance of the thymus in translational settings. Reconstituting the peripheral immune system after severe immune depletion such as chemotherapy, irradiation or other immune ablative events would benefit from increased thymus function to supply newly generated naïve T cells into the pool (65, 66). Without continuous thymic output, memory T cells would eventually outcompete and outnumber naïve T cells, resulting in diminished TCR diversity and compromising homeostasis of the peripheral T cell pool. Therefore, further investments to identify mechanisms that can rejuvenate the thymus or boost thymic output of naïve T cells are critical to replenish an immunocompetent and diverse peripheral T cell pool. Lastly, our current observations are in agreement with the seminal study by Surh et al. where they observed memory CD8 T cells to utilize either or both IL-7 and IL-15 for survival and homeostatic proliferation (67). However, our multipronged approaches of investigations on lymphopenia-induced proliferation under conditions of competition significantly expands the scope of our understanding, and now provide the molecular basis of distinct repopulation kinetics of naïve and memory T cells.

## Materials and Methods

### Mice

C57BL/6 (CD45.2) and C57BL/6 CD45.1 congenic mice were purchased from the Charles River Laboratories. *Rag2*^−/−^ mice were purchased from The Jackson Laboratory. *Rag2*^−/−^*Il15*^−/−^ mice were generated in house by breeding *Rag2*-deficient mice with IL-15-deficient mice. *Rag2*-GFP-Tg mice were previously described and obtained from the Jackson Laboratory (7). Mice with T cell-specific deletion of STAT5a/b were previously reported and maintained in house (68). Animal experiments were approved by the NCI Animal Care and Use Committee, and all mice were cared for in accordance with NIH guidelines.

### Flow Cytometry

Cells were harvested from the thymus, spleen, and lymph nodes. Data were acquired using an LSRII flow cytometer (BD Biosciences) and analyzed using FlowJo. Live cells were gated using forward scatter exclusion of dead cells stained with propidium iodide. Naïve and memory T cell subpopulations were electronically sorted using a FACSAria II (BD Biosciences) cell sorter, based on their GFP and CD44 expression levels. In brief, single cell suspensions were stained for TCRβ, CD4, CD8, and CD44 expression and resuspended in sorting buffer (0.5% BSA in Ca^2+^/Mg^2+^-free PBS) at 20 × 10^6^ cells/ml and filtered through 0.45 μm nylon meshes before passing through the cell sorter. Collected cells were washed once in PBS before further processing for tail vein injection or RNA isolation. The following antibodies were used for staining: TCRβ (H57-597), IL-7Rα (A7R34), CD44 (IM7), CD62L (MEL-14), CD4 (GK1.5), CD8α (53-6-7), and isotype control antibodies (eBioscience or BioLegend). Antibodies for pAkt (M89-61) and phosphor-mTOR (O21-404) were purchased from BD Biosciences, and used in staining kits from eBioscience following the manufacturer's instructions.

### Lymphocyte Isolation

Single cell suspensions were prepared from lymph node, spleen, liver and lung. Mononuclear cells (MNC) from liver and lung were prepared using lymphocyte isolation protocols as previously described with minor modifications (69). In brief, liver tissues were pressed through a 70 μm cell strainer (BD Biosciences) and resuspended in PBS. Cell suspensions were centrifuged at 100 g for 3 min, and supernatants were collected, spun down, and washed again with cold PBS. Liver samples underwent enrichment for lymphocytes by centrifugation in a two-step Percoll gradient (GE Life Sciences). Lymphocytes at the interphase were harvested, washed, and resuspended in cell culture media before further analysis. All liver MNCs were identified by expression of CD45.

### *In vitro* IL-7 Stimulation

T cells were stimulated with IL-7 as previously described (7). In brief, single cell suspensions were adjusted to 5 × 10^6^ cells/ml and stimulated with recombinant IL-7 (PeproTech) at 37 C for the indicated time. pSTAT5 contents were assessed after 30 min upon fixing and permeating cells with paraformaldehyde and acetone/methanol, followed by staining with anti-pSTAT5-specific monoclonal antibodies (clone 47, BD Bioscience).

### Cell Trace Violet (CTV) Labeling and Adoptive Transfer

Donor T cells were electronically sorted from lymphocytes isolated from LN, which were pooled out of inguinal, axillary, cervical, and mesenteric area. Before injection, donor cells were loaded with CTV (Invitrogen) as previously described (7). 10 × 10^6^ cells were tail-vein injected into *Rag2*^−/−^ or *Rag2*^−/−^*Il15*^−/−^ double deficient mice. Donor cells were recovered at indicated times, from spleen or lymph nodes for analysis.

### Statistical Analysis

Statistical tests were performed with Prism (GraphPad). Statistical significance was determined with Student's *t*-test. ^*P < 0.05 was considered significant. ^**^P < 0.01; ^***^^P < 0.001. ANOVA test was used to compare more than 3 groups of normally distributed data. Error bars indicate standard error of the mean (SEM).

## Author Contributions

HK designed, performed, analyzed experiments, and wrote the manuscript. HC, JK, EC, CJ, NH, and SH performed and analyzed experiments. J-HP and RG conceptualized the study, directed the experiments, analyzed data, and wrote the manuscript.

### Conflict of Interest Statement

The authors declare that the research was conducted in the absence of any commercial or financial relationships that could be construed as a potential conflict of interest.
