# Supplementary material for: Differential Cytokine Utilization and Tissue Tropism Results in Distinct Repopulation Kinetics of Naïve vs. Memory T Cells in Mice
Source: Front Immunol. 2019 Mar 4;10:355. doi: 10.3389/fimmu.2019.00355 (PMC6409349; doi:10.3389/fimmu.2019.00355)
Supplement: Supplementary file 1 [file Image_1.pdf]

Supplemental Figure 1

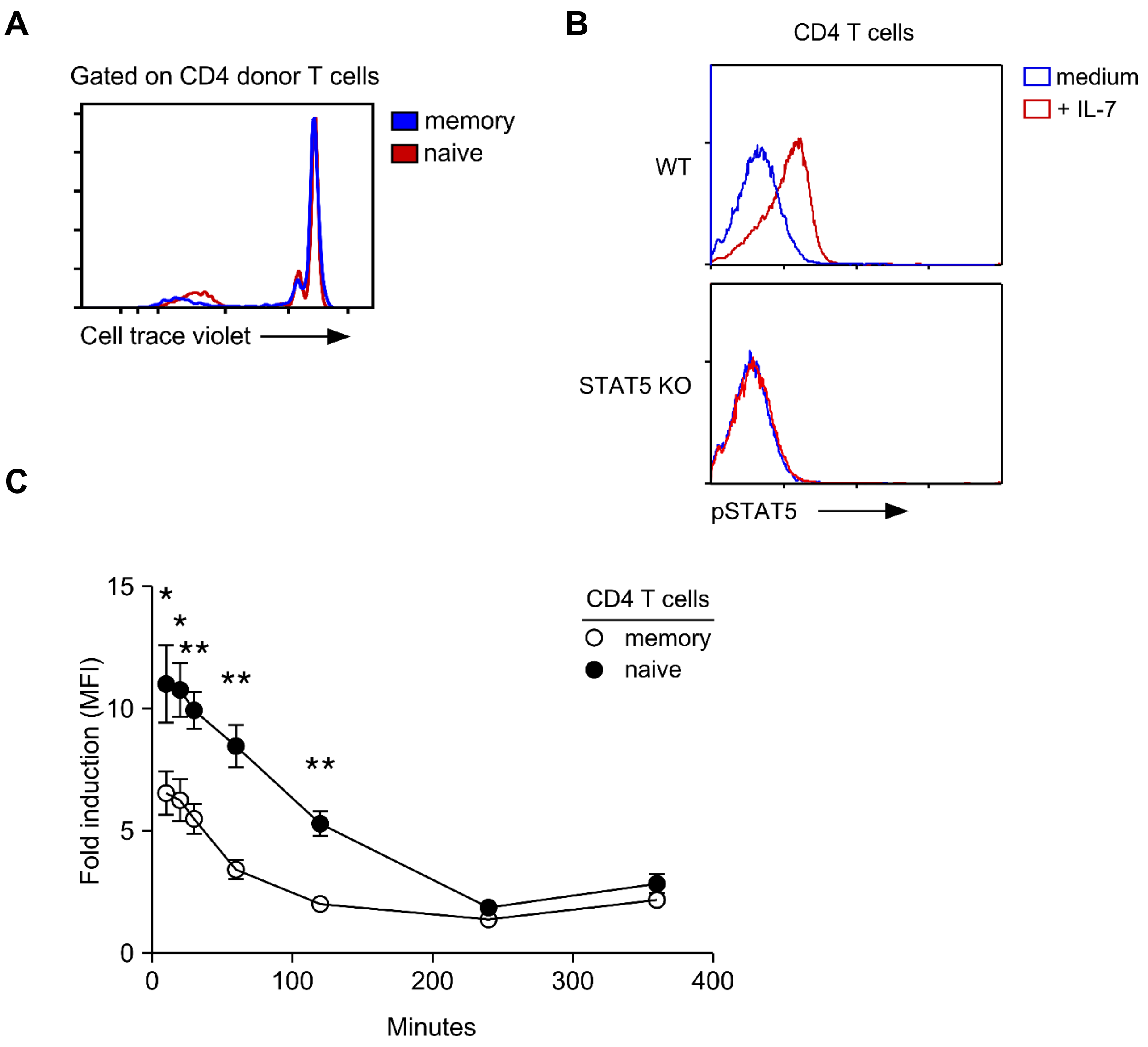

Supplemental Figure 1. Homeostatic proliferation and cytokine signaling of CD4 T cells

A. Cell trace violet dilution of memory versus naïve CD4 donor T cells that were adoptively transferred into *Rag2*-deficient host mice.
B. Intracellular pSTAT5 contents in IL-7-stimulated (1 ng/ml) WT and STAT5-deficient (STAT5-KO) CD4 T cells.
C. Kinetics of STAT5 phosphorylation upon IL-7 signaling (1 ng/ml) at indicated time points in naïve and memory CD4 T cells. Graph shows summary of two independent experiments. *, $P<0.05$; **, $P<0.01$

Supplemental Figure 2

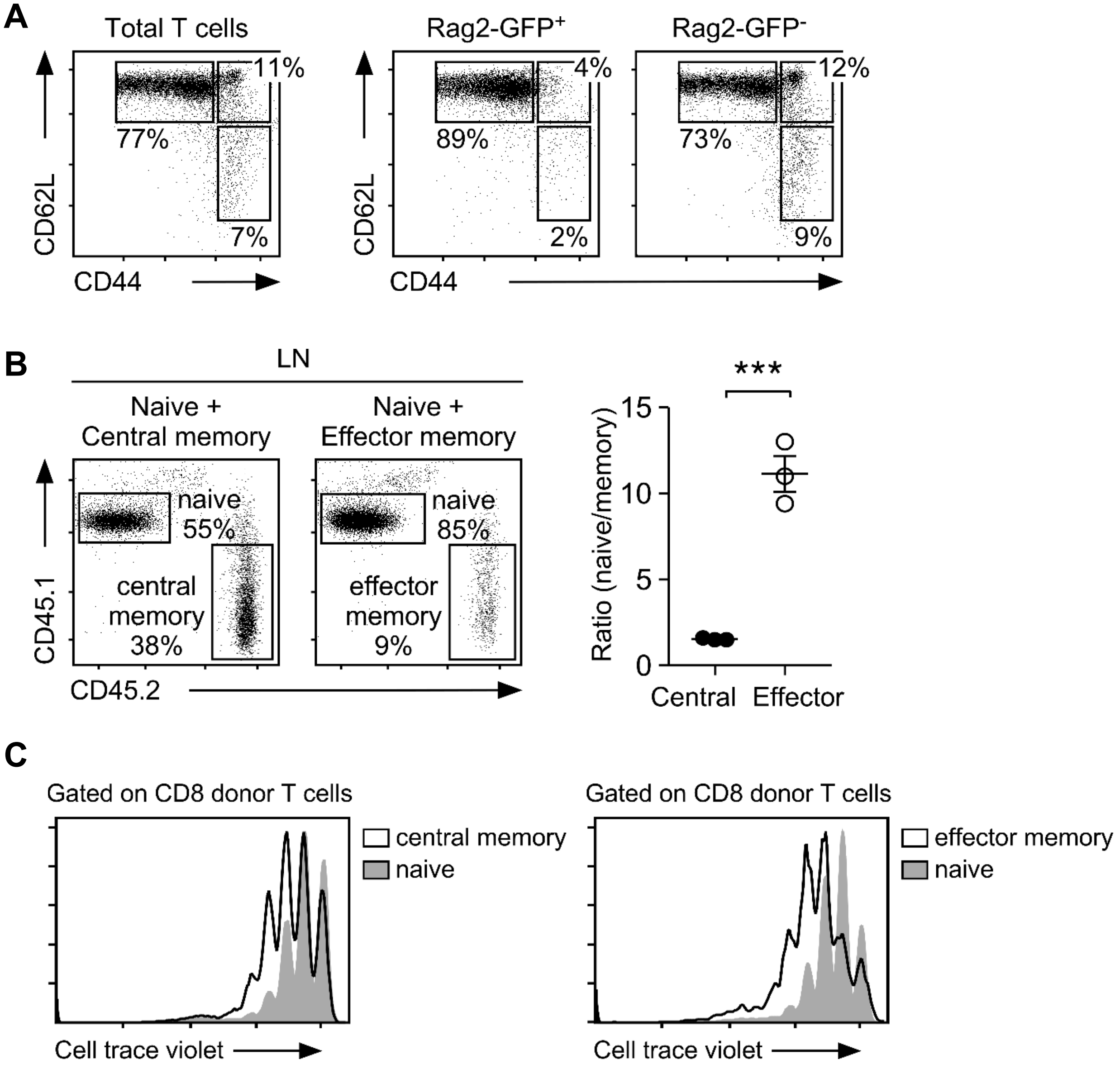

Supplemental Figure 2. Homeostatic proliferation of memory T cell subsets

A. T cell subsets among LN T cells based on CD62L and CD44 expression. Naïve cells are CD62L^{+}CD44^{low}, central memory cells are CD62L^{+}CD44^{hi}, and effector memory cells are CD62L^{-}CD44^{hi} (left). CD62L and CD44 expression in *Rag2*-GFP^{+} (RTE) and *Rag2*-GFP^{-} T cells (right).

B. Naïve (CD45.1) and congenic central or effector memory (CD45.2) T cells were injected into *Rag2*-deficient hosts and recovered 5 days later from the LN. Naïve versus central memory (left) or naïve versus effector memory (right) ratio after adoptive transfer. Results are summary of three independent experiments. ***, $P<0.001$

C. Cell trace violet dilution of central memory versus naïve (left) and effector memory versus naïve (right) donor T cells adoptively transferred into *Rag2*-deficient host mice.
